# Supplementary material for: Rapid turnover of life-cycle-related genes in the brown algae
Source: Genome Biol. 2019 Feb 14;20:35. doi: 10.1186/s13059-019-1630-6 (PMC6374913; doi:10.1186/s13059-019-1630-6)
Supplement: Supplementary file 3 — Protein alignments, corresponding Gblocks html file and CDS sequences for the lineage conserved gametophyte-biased genes. (ZIP 2109 kb) [file 13059_2019_1630_MOESM3_ESM.zip › Con_GA_alignments/Ec-01_007070.aa_ali.fasta-gb.htm]

Ec-01\_007070.aa.fasta.trans.aa\_ali.fasta


## Gblocks 0.91b Results

Processed file: **Ec-01\_007070.aa.fasta.trans.aa\_ali.fasta**  
Number of sequences: **2**  
Alignment assumed to be: **Protein**  
New number of positions: **587** (selected positions are underlined in blue)

```
                         10        20        30        40        50        60
                 =========+=========+=========+=========+=========+=========+
Ec-01_007070     -------------------MVERVNDVRKEDVKQRTLRITWKAERPTDEELLDAFRPLGD
MSTRG.43673.1    RAESYTSPEHSFNSTYLVFLLRRYSSAHATHRRMCAKSPACQTPVFMHISLFVLCACRSD
                                                                   ##########


                         70        80        90       100       110       120
                 =========+=========+=========+=========+=========+=========+
Ec-01_007070     VVR-----------------VQAKERCAAILFASAEPVPAAVAFATAAVEDSPWNNWDVQ
MSTRG.43673.1    LFCNNSTVSYACHHGRPFHVNVTQDKCAAVLFASAESVAAAV----AAVQDGPGRTWRVQ
                                           ################    ##############


                        130       140       150       160       170       180
                 =========+=========+=========+=========+=========+=========+
Ec-01_007070     SMAG-----------------------------IVHITNEMSKLSRETSDDEGPQADVNG
MSTRG.43673.1    PMADTRQKSKENVRRLIAGGRSRSWAASTGRPEVTAISISMSGTTERKDASEPGLAEQHQ
                 ###                                                         


                        190       200       210       220       230       240
                 =========+=========+=========+=========+=========+=========+
Ec-01_007070     RKH---RN-------------TIPQDAPIDQERGSMTRECVEISSGTKDTAPAMDKVQNN
MSTRG.43673.1    DSQREEGEIIIEEAGACCAKRSLRKGDVDKEPRSTGRAPTEHQISKAKRTVSPLNQLQRE
                                                             ################


                        250       260       270       280       290       300
                 =========+=========+=========+=========+=========+=========+
Ec-01_007070     SRQRRESKTRSE----------------SNSGGALEGQRQREEDTQKHVEEKTPSPREGV
MSTRG.43673.1    SNVLFLGKSRSVPAASSSSSDSGNEELFSSTGGDCRHCRQNDQQRLKTKSSRWGLLTEDA
                 ###########                 ###################             


                        310       320       330       340       350       360
                 =========+=========+=========+=========+=========+=========+
Ec-01_007070     TSHSSPRANDALRDASDMVRQEPSPLSSPEGLVAPPMWEEDKG-VELGETIDVDG--AHG
MSTRG.43673.1    LRKQQQQYLPGVQKSTERVKTRKSSPKDVHGSDDSAMCRQEPSPFSLPEELRAWSPCTQT
                                   ###################                       


                        370       380       390       400       410       420
                 =========+=========+=========+=========+=========+=========+
Ec-01_007070     ARGSGSNYGGGGDTSCPRTPGDVRADASGAGDDDPAPQLLHAGEAAPLATSKRPEVATCQ
MSTRG.43673.1    RDHDGLKLKTNDEKKTGEHKGGLTHGEACTKTDGGPASSVGGDAVPPRSASDEPHLATD-
                                                               ############  


                        430       440       450       460       470       480
                 =========+=========+=========+=========+=========+=========+
Ec-01_007070     QPEDRIHSTSGMKTSSAPAATTDNVKAAGTNVTSNEQDILTARSGNYSLERGDETWASEH
MSTRG.43673.1    ---RRVHGQQQNQERSAKRASLDGNKSGGTDRESAKKGDTTTHGGDELPGRSDKAEAPDK
                     ########################################################


                        490       500       510       520       530       540
                 =========+=========+=========+=========+=========+=========+
Ec-01_007070     QKT--RDQAKRLALEVARLRSSLRTTTSELNTERSTRVRLEDEWWRRMQEWEEERGRLVA
MSTRG.43673.1    QQAKQTKQAKRLALEVARLRSSLRATTSELHAERSARSRLEDEWWKKTRAWEEERSSLVT
                 #      #####################################################


                        550       560       570       580       590       600
                 =========+=========+=========+=========+=========+=========+
Ec-01_007070     SKQEAERTARTAEEDVARLLVKEKDREGEVASERLARETRQEKADIAMNGVVKVQDRLIQ
MSTRG.43673.1    STQNAERASKLAQEEVGRALAKGKAREVEITSERVVREAAQGKTESALKGVVNVQDRLLG
                 ############################################################


                        610       620       630       640       650       660
                 =========+=========+=========+=========+=========+=========+
Ec-01_007070     ALRTELEMTDRARQAAQDNALEAKDQLRLYVDDFLLWRNRAKHLEGLLIDLGHPPQPGLN
MSTRG.43673.1    ALQSELEMTNRAREAAQDDSREAKEQLHLYVQDFLLWRDRAKRLGAREVQAGCPAHSKRH
                 ############################################################


                        670       680       690       700       710       720
                 =========+=========+=========+=========+=========+=========+
Ec-01_007070     TETPLSCGSPSSKEHKLPEAPHRQNVDDKEQEADSRTPVAGEPVEYGGSRQAGDGAKGLS
MSTRG.43673.1    AEAPPACDSPS-------EPLQQRAADDIQRTTGAKDEDKSIEHASSAQAREPEVSSDLS
                 ###########       ##########                              ##


                        730       740       750       760       770       780
                 =========+=========+=========+=========+=========+=========+
Ec-01_007070     IPEKYARYFETTTGGHPAPPSNPAFPGTQRTEASELDSETAAAAAAWSTRAAMAESSSSS
MSTRG.43673.1    IPEKYARYFETTTGGCASAAPSTEPVRLRQGDTPEGEPEAAAAAAAWSTRSAMAG-ALSS
                 ###############                   ####################    ##


                        790       800       810       820       830       840
                 =========+=========+=========+=========+=========+=========+
Ec-01_007070     SSSRAYFHRSMKAQQEISIRLGKDLARARADLQSIYESNKAKARRGGGETSTCHGSLLHE
MSTRG.43673.1    PSSRAYFQRSMKAQQEISHRLERDLVRARADLQAIYAGSRTRSSGESRDERATSLASLPE
                 ####################################                        


                        850       860       870       880       890       900
                 =========+=========+=========+=========+=========+=========+
Ec-01_007070     TDGKSEAASSERAQGIGTGRNGGESPSDERRVQDRTAVQGVSAP---------------A
MSTRG.43673.1    EAGCSLVDPVRNEAQGSTRTEKNRDDDSQAREESCQILEGVDPDAGDAPDQQVVPDAAAC
                                               ###########                   


                        910       920       930       940       950       960
                 =========+=========+=========+=========+=========+=========+
Ec-01_007070     ASFPEGSHMARVLAVLLGEDSTAN-GTAVAGLSDAPTPSLRLTDYPQMLQAVFGGGPAAP
MSTRG.43673.1    TSVARHSYTGRVLAALLGGDLAASPRTTASGLPGASIRTLRLSDYPPALQAVFSEWPAAV
                  ######################   ################################# 


                        970       980       990      1000      1010      1020
                 =========+=========+=========+=========+=========+=========+
Ec-01_007070     --LPDSAVTHRRRVVVSEAPQRGQTGQGLYGDDDEGRFPTQHPSRSRTLTEEKQQARTTQ
MSTRG.43673.1    PLPPAPAASPNRRLRGAEAPH-----------HDRSRVPSRSPSWRAAPHTDENEGREQE
                    #################             ###########            ####


                       1030      1040      1050      1060      1070      1080
                 =========+=========+=========+=========+=========+=========+
Ec-01_007070     SHARVDRRHGPGATGRNCTEVQVHNRTCGGNGGRGDAGCRPQDKAAVEGASGIPDDVDPG
MSTRG.43673.1    SLIGVDKNGSPPAKNAAALVAGENSSSSADANAAGKTSGVLGARGSDHQSADMATAAEGM
                 #############                                               


                       1090      1100      1110      1120
                 =========+=========+=========+=========+===
Ec-01_007070     GGESRSLDRAFRGYEAKSSLLQRWLGEKKSPSPSPSRAMPVT-
MSTRG.43673.1    RG-GCSVSGAFRGYEAKSPFLRRWLGERFSPSPQPRMTAIEQN
                      ##############################
```

```
Parameters used
Minimum Number Of Sequences For A Conserved Position: 2
Minimum Number Of Sequences For A Flanking Position: 2
Maximum Number Of Contiguous Nonconserved Positions: 8
Minimum Length Of A Block: 10
Allowed Gap Positions: None
Use Similarity Matrices: Yes
```

```
Flank positions of the 20 selected block(s)
Flanks: [51  60]  [87  102]  [107  123]  [225  251]  [269  287]  [319  337]  [407  418]  [425  481]  [488  671]  [679  688]  [719  735]  [755  774]  [779  816]  [871  881]  [902  923]  [927  959]  [964  980]  [994  1004]  [1017  1033]  [1086  1115]  

New number of positions in Ec-01_007070.aa.fasta.trans.aa_ali.fasta-gb:  587  (52% of the original 1123 positions)
```
